# Mucoadhesive Delivery System: A Smart Way to Improve Bioavailability of Nutraceuticals

**DOI:** 10.3390/foods10061362

**Published:** 2021-06-11

**Authors:** Parthasarathi Subramanian

**Affiliations:** Riddet Institute, Massey University, Private Bag 11222, Palmerston North 4442, New Zealand; P.Subramanian@massey.ac.nz

**Keywords:** bioavailability, mucosal delivery system, nutraceuticals, bioactive compounds, films, wafers, 3D printing, personalized formulation

## Abstract

The conventional oral administration of many nutraceuticals exhibits poor oral bioavailability due to the harsh gastric conditions and first-pass metabolism. Oral mucosa has been recognized as a potential site for the delivery of therapeutic compounds. The mucoadhesive formulation can adhere to the mucosal membrane through various interaction mechanisms and enhance the retention and permeability of bioactive compounds. Absorption of bioactive compounds from the mucosa can improve bioavailability, as this route bypasses the hepatic first-pass metabolism and transit through the gastrointestinal tract. The mucosal administration is convenient, simple to access, and reported for increasing the bioactive concentration in plasma. Many mucoadhesive polymers, emulsifiers, thickeners used for the pharmaceutical formulation are accepted in the food sector. Introducing mucoadhesive formulations specific to the nutraceutical sector will be a game-changer as we are still looking for different ways to improve the bioavailability of many bioactive compounds. This article describes the overview of buccal mucosa, the concept of mucoadhesion and related theories, and different techniques of mucoadhesive formulations. Finally, the classification of mucoadhesive polymers and the mucoadhesive systems designed for the effective delivery of bioactive compounds are presented.

## 1. Introduction

Nutraceutical, a hybrid term for denoting “nutrition” and “pharmaceutical”, was coined in 1989 by Stephen L. DeFelice, founder and chairman of the Foundation for Innovation in Medicine (FIM). DeFelice defined nutraceutical as “any substance that is a food or a part of the food and provides medical or health benefits, including the prevention and treatment of disease” [1,2]. Recently, nutraceuticals have gained much attention for the benefits of reducing lifestyle-associated diseases, including arthritis, asthma, type II diabetes, obesity, cardiovascular disease, and hypertension. However, the nutraceutical compound may possess poor physical stability, permeability, and bioavailability, which limits the absorption from the GI tract. This poor absorption pattern of conventional nutraceutical formulation put pressure on the research scientist to deliver nutraceutical/bioactive products more bioavailable through oral administration [3].

Among many administration routes of therapeutic formulation, consumers prefer oral administration as it does not need any supervision. However, the oral route of administration of nutraceuticals possesses major disadvantages such as degradation of therapeutic activity by gastrointestinal enzymes and first-pass metabolism, leading to poor bioavailability [4]. The oral mucosal delivery system is a widely accepted novel administration route for pharmaceutical formulations. Thus, the oral mucosal delivery system is appreciated for avoiding the first-pass metabolism and enzymatic degradation in the GI microflora and achieves immediate and controlled release action. This review is aimed to narrate the application of mucoadhesive dosage in nutraceutical aspects that might be a useful tool for designing novel mucoadhesive delivery systems.

## 2. Mucoadhesion

The mucoadhesion concept has attracted much attention in the pharmaceutical sector and is effectively used as a route of administration. The mucus membrane (also called mucosa) is a moist tissue lining that covers the organs and cavities such as the mouth, nose, eyelid, gut, and rectum. Leung and Robinson [5] describe mucoadhesion as the interaction between a mucous surface and a synthetic or natural polymer. The polymer carrier containing therapeutic material will adhere to the targeted mucosa for an extended period, thereby increasing its permeation and bioavailability [6]. Many readers may confuse the term “mucoadhesion” with “bioadhesion”. In mucoadhesion, the polymer is attached to mucus surface (substrate), whereas in bioadhesion, the polymer is attached to the biological surface (it may be epithelial tissue or mucus coat on the surface of the tissue) [7]. Oral mucosal delivery is further classified into three categories: (i) sublingual delivery, systemic delivery of therapeutic compounds through the mucosal surface of the mouth; (ii) buccal delivery, administration through the mucosal linings of cheeks (buccal mucosa); and (iii) local delivery, administration through the oral cavity. The buccal mucosa is widely applicable for drug administration, and sublingual delivery is useful for the fast onset of therapeutic action (ex: sublingual nitroglycerin for the treatment of Angina pectoris).

### 2.1. Oral Mucosa

**Figure 1:**
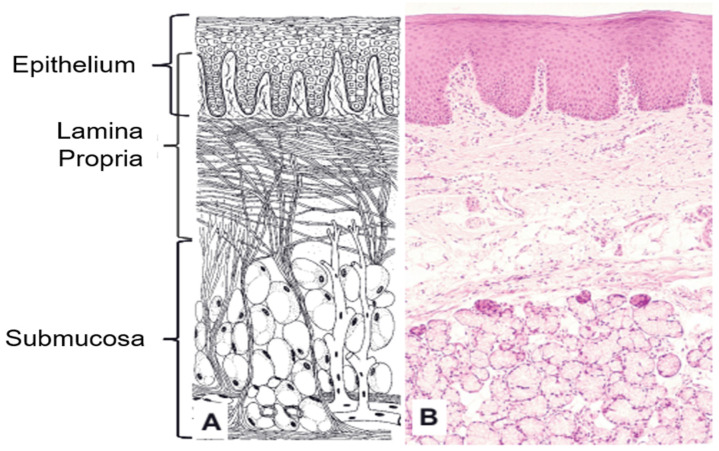
(**A**) Schematic diagram of the oral mucosa; (**B**) histological section of the hard palate to show the tissue components (Reprinted with permission from Chen et al., 2015 [9]).

A deep understanding of the histology of mucosa is necessary for fabricating the mucoadhesive nutraceutical formulation. Oral mucosa is a moist membrane lining on the surface of the oral cavity apart from teeth. It occupies a total surface area of 200 cm^2^ and consists of two anatomical and functional layers: (i) stratified squamous epithelium at the outermost layer, and (ii) an underlying basement membrane of mesodermal origin, lamina propria (see Figure 1) [8].

The epithelium (a protective layer) of the oral mucosa comprises approximately 40–50 cell layers thick, and it is divided into the nonkeratinized and keratinized epithelium. The differentiation of keratinized and nonkeratinized epithelia is due to the presence or absence of a cornified surface layer. Keratinized epithelium is found in the hard palate and non-flexible regions (subject to mechanical stress) of the oral cavity, containing neutral lipids (ceramides and acylceramides) for barrier function. In contrast, nonkeratinized epithelium found in the soft palate, sublingual, and buccal region, originating from the basal cells, containing a small amount of neutral polar lipids (cholesterol sulfate and glucosyl ceramides) with a lack of acylceramides [10,11]. These nonkeratinized epithelia received considerable attention among researchers for delivering their therapeutic formulation for their low enzymatic activity, highly vascularized, and permeability characteristics than keratinized epithelia [12]. Pramanik et al. [13] measured the thickness and protein concentration of mucosal fluids at four mucosal surfaces (anterior hard palate, buccal mucosa, anterior tongue, and lower labial mucosa). Anterior hard palate and the labial mucosa were reported for thin mucosal surface and high concentration of protein (Table 1).

Oral mucosa was reported for its high deformation under compression and exhibited dynamic response over time under loading and unloading, attributed to the fluidic components within the mucosa matrix. Stiffness of the mucosa is attributed to both the solid matrix structure (e.g., epithelial layer, fibrous network, blood vessel, etc.) and fluid components (e.g., interstitial fluid, blood) [9]. Further, the permeability of buccal mucosa is 4–4000 times greater than that of skin [14], and there is a considerable difference in permeability between regions of the oral cavity in the order of sublingual > buccal > palatal [15]. The permeability barrier function is responsible for protecting endogenous and exogenous molecules. Permeability of oral mucosa is ensured by the intercellular spaces of superficial epithelial layers and submucosa. The intercellular lipid content is migrated to the apical cell surface to fuse with cell membrane and the lipid content is discharged into intercellular space to form a barrier of 200 µm superficial layer [11]. Marxen et al. [16] reported that epithelium acts as a strong barrier for the permeation of nicotine and mannitol across the porcine buccal epithelium and buccal submucosa. Nicotine permeability at submucosa (P_app,submucosa_ = 2.41 × 10^–5^ ± 0.17 × 10^–5^ cm/s; thickness = 635 ± 55 µm) was significantly higher than the epithelium (P_app,epithelium_ = 1.20 × 10^–5^ ± 0.24 × 10^–5^ cm/s; thickness = 423 ± 46 µm), irrespective of the thickness. On the other hand, the submucosal permeation of mannitol (P_app,submucosa_ = 1.96 × 10^–5^ ± 0.16 × 10^–5^ cm/s) is similar to nicotine, however the permeation across epithelium is negligible. These findings revealed that epithelium contributes a major barrier property for the permeation of drugs and the requirement of choosing the right mucoadhesive enhancers for effective delivery.

### 2.2. Absorption Mechanism

**Figure 2:**
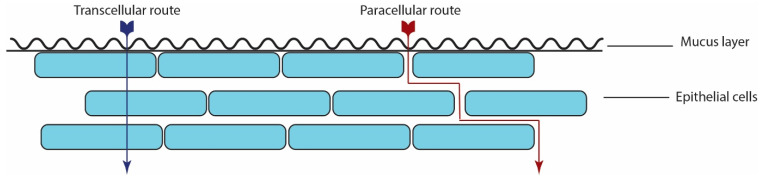
Transport pathways in the oral mucosa.

Oral epithelial cell membranes are lipophilic; however, the space between the epithelial cells is hydrophilic and results in hydrophilic and lipophilic regions. Accordingly, the penetration of drugs across the oral mucosa follows a passive diffusion process by paracellular route (transport of drug through the intercellular spaces between the cells) and transcellular route (transport of molecule across the cells). Figure 2 depicted the transport pathways in the oral mucosa. Researchers suggested that hydrophilic molecules choose a paracellular route, and lipophilic molecules preferentially penetrate through the transcellular route [17,18]. Fick’s law of diffusion can be applied for the drug absorption process (Equations (1) and (2)):

(1) $$P=\frac{D\cdot K_{p}}{h}$$

(2) $$A=P\cdot C\cdot S\cdot t=\frac{D\cdot K_{p}}{h}\cdot C\cdot S\cdot t$$

where P, D, K_p_ are permeability coefficient, diffusion coefficient, and partition coefficient of a drug in the mucosal formulation to the oral mucosa, respectively. “A” is the amount of drug absorbed, “C” is the free drug concentration in the delivery medium. “S” and “t” are the surface area and duration time of formulation contacting the oral mucosa [19].

Lipid matrix between the extracellular space act as an important barrier for the paracellular pathway, especially for high molecular weight compounds. Recently, [20] revealed the increase in drug release from the buccal films at lower pH attributed to the faster release characteristics of organic acid from the buccal films. Further, the ability of a drug to penetrate the oral mucosa depends on the lipid solubility, denoted by the oil/water partition coefficient [21]. Commercial formulations for oral transmucosal delivery have a log *p* valve (octanol/water) above 2.0, representing the drugs are 100 times more readily soluble in octanol than in water. At the same time, the drug with suitable water solubility allows the drug to diffuse across the cytoplasm (hydrophilic) of the cells. However, highly lipophilic drugs tend to have poor water solubility, which brings the concept of the pKa value of a drug. The pKa value of the drug determines the degree of ionization of the drug at different pH. The ideal candidate for buccal delivery needs to be unionized at the site for better absorption [22].

### 2.3. Mucoadhesion Theories

**Figure 3:**
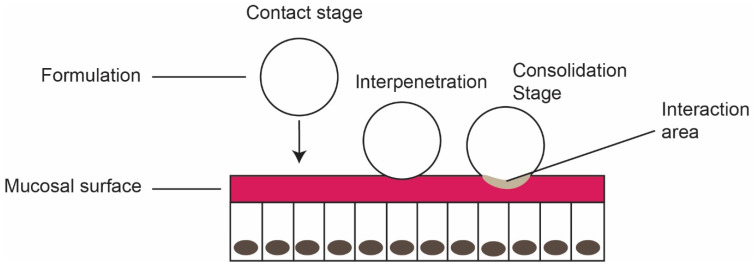
Stages of Mucoadhesion (Reprinted with permission from Smart, J.D, 2005 [23]).

Mucoadhesion is a complex process initiated from the connection between mucoadhesive material (formulation) and mucous membrane by three stages [23,24]:

1. Contact stage: The first stage in the mucoadhesion, initiating the contact between mucoadhesive formulation and the mucous membrane. Wetting and/or spreading of the material enhances the contact stage and increases the surface area.
2. Interpenetration stage: Diffusion of mucoadhesive polymer into the mucus layer through spreading and deep contact with the mucus layer.
3. Consolidation stage: Strengthening of mucoadhesive joints through mechanical and/or chemical interactions for prolonged adhesion (see Figure 3). Mechanical bonds are physical interactions relating to the penetration of mucoadhesive polymer into the mucus layer. Chemical bond includes strong primary bond and weak secondary bonds, based on the polymer structure.

Various theories are proposed to explain the mucoadhesive phenomenon. However, many theories were failed to explain the diverse range of adhesive interactions. The following theories were widely accepted by formulation scientists:

- Mechanical interlocking: According to this theory, adhesion is by interlocking the adhesives into the rough surface. Such irregular surface offers a higher surface area available for interaction between the adhesive and mucus [25];
- Electronic theory: The formation of an electrical double layer at the adhesive-mucus interface due to electron difference between adhesive and mucus layer facilitates the attractive force [26];
- Diffusion theory: Also called interpenetration theory. Adhesive material penetrates in-depth into the mucus layer and creates a semi-permanent adhesive layer. The penetration depth of adhesive polymer depends on the molecular weight (polymer chain length) and diffusion coefficient. The adhesion will not be a simple two-dimensional surface phenomenon; it will be a three-dimensional process [27];
- Adsorption theory: Adhesive material sticks to the surface through hydrogen bonding, van der Waals, and hydrophobic interactions. Though they are secondary weak forces, the sheer number of interactions provides intense adhesive strength [26];
- Wetting theory: This theory applies to the liquid adhesives, considering the interfacial tensions to predict spreading and adhesion [23];
- Fracture theory: Above theories were developed based on the joining behavior of adhesive material with the mucus layer. Fracture theory defines the force required to detach after adhesion, and the fracture is assumed to occur at the mucoadhesive interface. Further, the fracture strength strongly depends on the length of the polymer chain and the degree of cross-linking [28].

Apart from the above-mentioned theories, chemical interactions such as electrostatic, hydrophobic, hydrogen bonding, etc., play a crucial role in the mucoadhesion phenomenon.

- *Electrostatic interactions* (also called as van der Waals interaction) appear if the charges (either positive or negative) are separated by a distance due to ionization or attachment of ionic species. Sialic acid and ester sulfates in the mucus layer provide a negative charge, which creates strong electrostatic interaction with the positively charged mucoadhesive polymers such as chitosan [29]. On the other hand, the negatively charged molecules (acrylates) can affix with mucins through the positively charged amino acids in the terminal domains;
- *Hydrophobic interactions:* The hydrophobic interactions can form between the naked protein core of mucin or lipids of mucus and the diffusion compounds created between the mucus and diffusing drugs. The hydrophobic interaction plays a key role in the tail-to-tail aggregation of mucins. For effective hydrophobic interaction to occur, it requires high energy and low sensitivity to the surrounding conditions. Thus, hydrophobic interaction is effective in gastric conditions where pH is very low and suppresses the electrostatic interactions [30].

## 3. Mucoadhesive Dosage Formulations

The oral mucoadhesive delivery system includes tablets, film and patches, semisolids/liquids, and particulates. Unlike the oral therapeutic formulation, mucoadhesive dosage forms need to address a list of challenges presented in Table 2 [31].

### 3.1. Buccal Tablets

Buccal tablets are the first choice of formulation scientists for the delivery of therapeutic compounds. Buccal tablets are compact, small, oval with the size of approximately 5–8 mm diameter [32]. The main advantage of this tablet is, it can be placed/applied at different regions in the oral cavity including, the palate, cheek mucosa, and the region between upper lip and gum. These buccal tablets are aimed to retain at the same position until there is a complete dissolution and/or complete release from the formulation. The retention time of the buccal tablets can be triggered between 4–6 h and 7–12 h depending on the oral location [26]. The major disadvantage of the buccal tablets includes the acceptability (children, elderly, and people wearing dentures may feel discomfort), detachment of tablet from the mucosa, migrating to the esophagus, and swallowing. The buccal tablets are usually produced by direct compression technique, so it is important to understand the pre- and post-compression parameters. Pre-compression parameters mainly include the flow properties including tapped and bulk density and the related Carr’s Index and Haunser’s Index. Angle of repose is an important pre-compression parameter used for storage and conveying system of particulate matters [33]. If the “angle of repose” is high (>55 degrees), it represents the particulates are sticky, and the low “angle of repose” (<30 degrees) represents the smooth and spherical particulate. Thus, lower the “angle of repose”, it is easier for the material to travel with little energy or even by gravitational force [33]. Post-compression parameters include thickness (maintain uniformity in each tablet), hardness (withstand physical/mechanical stress), mucoadhesion strength (how effective the formulation adhesive with the mucosal surface), swelling index, retention time, dissolution studies (release characteristics), diffusion study (nutrient diffusion into the mucosa) and pharmacokinetic study. Post-compression parameters are tested in vitro (physical test such as thickness, hardness, etc.), ex vivo (goat mucosa is used for studying the mucoadhesive strength and retention time), and in vivo (pharmacokinetic study in animals).

Gowthamarajan et al. [34] developed the curcumin buccal tablets using natural gum from a cashew nut tree. The authors evaluated the buccal residence time and buccal acceptance for the placebo buccal tablets (without curcumin) with human volunteers and studied the comfort, acceptability, salivation, irritation, and disintegration. The authors found that by increasing the concentration of mucoadhesive polymer (cashew nut tree gum) there is a significant increase in mucoadhesive strength. Increasing the concentration of cashew nut tree gum, it forms a secondary bioadhesion bond with mucin and undergoes extensive interpenetration with the mucus layer. In a recent study, Mohamad et al. [35] developed buccal vitamin B12 tablets by direct compression and employed different concentrations of hydroxypropyl methyl cellulose (HPMC), Carbopol (CP), and chitosan (Cs). Carbopol (CP) is a trademark owned by Lubrizol Corporation, USA. Carbopol is a high molecular weight, hydrophilic acrylic acid polymer, widely used for its aqueous solubility, biodegradability, and bioadhesive property [36]. Bioadhesive strength of Carbopol-based buccal tablets showed higher bioadhesive strength of 150 ± 0.5 mN (90% Carbopol in the formulation). Authors revealed the sustained release characteristics of vitamin B12 from buccal tablets and a 2.7-fold increase in bioavailability of vitamin B12 buccal tablet formulation than that of intramuscular administration. It was surprising to see that the C_max_ of I.M administered rabbits with 109.29 ± 9.39 pg/mL at 15 min, 43.23 ± 2.034 pg/mL at 30 min, and decreased gradually. On the other hand, the plasma concentration of buccal formulations showed a gradual increase in C_max_ till 30 min (40.25 ± 5.23 pg/mL) and decreased with a constant rate. Area under the curve (AUC) data are quite helpful in analyzing the bioavailability properties; AUC for buccal formulation and injection was 35706 ± 1.375 pg mL^−1^ min^−1^ and 13842 ± 1.689 pg mL^−1^ min^−1^, respectively. It is important to note that the polyanionic polymer (Carbopol) contains many carboxylic groups and provides strong H-bonding with mucosa and achieves better mucoadhesion. In addition, Carbopol-based formulations increase the viscosity after contacting with saliva and lead to sealing of the surface pores and thereby preventing the rapid release. Some of the notable research work on different mucoadhesive dosage formulations related to nutraceutical ingredients and their key findings are presented in Table 3.

### 3.2. Buccal Films

Buccal films are a thin, flexible sheet of material composed of polymer, therapeutic compounds, sweetener, and flavor [53]. Buccal films are the potential formulations for the effective delivery of nutraceuticals due to their versatility and flexibility. Further, these mucoadhesive films can remain in contact with the oral mucosa and provide prolonged release in the direction of the oral mucosa or toward the oral cavity [54]. Food and Drug Administration (FDA) defined three different types for films [55]:

- Film: A thin layer or coating;
- Film for extended-release: Films releasing the embedded therapeutic compounds over an extended period and maintains the constant level in the blood or target tissue;
- Film, soluble (also called orodispersible films): Thin layer or coating, being dissolved when in contact with saliva.

Mucoadhesive buccal films are manufactured by the following methods: solvent casting method, hot-melt extrusion, and printing method.

#### 3.2.1. Solvent Casting Method

**Figure 4:**
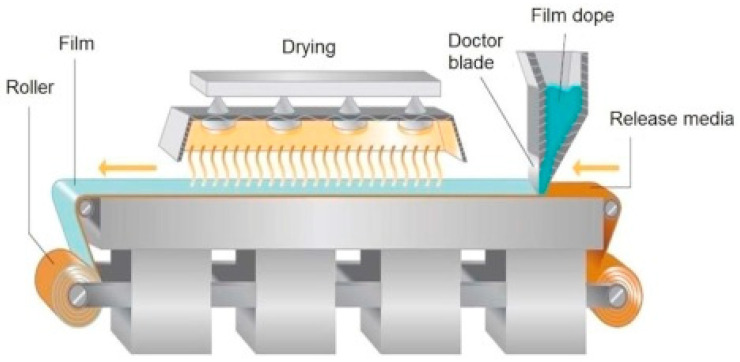
Manufacturing process of buccal films by solvent casting method (Reprinted with permission from Karki et al. 2016 [57]).

Solvent casting is the most widely used technique for the preparation of films due to the simple process, and it can be fabricated at a laboratory scale. The manufacturing process of buccal films involves three steps [55]: (i) preparation of the homogenous mixture of components containing bioactive compounds, mucoadhesive polymers, taste-masking compound, permeability enhancer, and plasticizers in an appropriate solvent; (ii) casting the resulted suspension/solution on molds to assure constant thickness of the film and uniformity of bioactive content; and (iii) drying and cutting of the casted film containing the desired amount of formulation. During the manufacturing process, rheological properties, uniformity of bioactive compounds, and residual solvents in the final dosage form are the critical steps for film performance. Because rheology determines the drying rates and the presence of organic solvents possessing undesired hazards for health. For this reason, researchers prefer to use water as a solvent during the manufacturing process. It is important to note that the introduction of air bubbles during the mixing of components (step 1 of the manufacturing process) and leads to an uneven surface with heterogeneous thickness. Removal of air before casting is a crucial step for maintaining homogeneity [56]. In addition, the translation of buccal film production from lab scale to production scale is one of the bigger challenges because of the involvement of many unit operations, including heating and mixing. Figure 4 depicted the commercial machine used for the production of buccal films based on solvent casting technique.

#### 3.2.2. Liposomal Buccal Film

Liposomes are lipid-based vesicular systems and recognized as an effective delivery system of nutraceuticals/bioactive compounds. Further, lipid-based delivery systems are reported for (1) better protections, (2) control release characteristics, (3) biodistribution, (4) targeted delivery, and (5) improving solubility and bioavailability [58]. Combining the two techniques viz. liposomes and mucoadhesive buccal delivery will address both solubility and permeability of a bioactive compound. Abd El Azim et al. [59] reported the prolonged release of a class 3 BCS (Biopharmaceutics Classification System) compound (vitamin B6-VB6) possessing high water solubility and poor permeability by combining two techniques: (1) liposomal formulation of vitamin B6 (improve the permeability) and (2) formulation of buccal films from the liposomal formulation (improve the residence time and release profile). The formulated VB6 buccal films possess the mucoadhesive strength of 20.55 ± 0.2 g for 0.2 N force of adhesion and able to attach in the buccal mucosa for 4.43 ± 0.07 h in three human volunteers. Liposomal buccal films reduced the cumulative permeated amount of vitamin B6 corresponding to the liposomal vitamin B6 formulation (1.2 times higher than the buccal films). Similarly, there is 36.89% reduction in flux between the liposomal buccal film (113.10 μg cm^−2^ h^−1^) than the liposomal formulation (179.20 μg cm^−2^ h^−1^).

In mucoadhesive delivery systems, various temperature-responsive materials have been employed during the manufacturing process. These thermoresponsive polymers such as poly(*N*-isopropylacrylamide (PNIPAAm) derivatives, poly(ethylene oxide)-poly(propylene oxide) (PEO–PPO) pluronic copolymers) can be triggered by a small variation in temperature. Pluronic copolymer (also called poloxamer) consists of blocks of hydrophilic PEO and hydrophobic PPO blocks in the form of A-B-A triblock structure, which turns liquid into gel form at physiological temperature [60]. These thermoresponsive characteristics of poloxamer help in preventing the formulation to be removed from the oral cavity due to mucociliary clearance [61]. A combination of bioadhesive polymer (Carbopol) and thermoresponsive polymer (poloxamer) is a suitable choice for buccal film formulation. In a recent study, curcumin buccal film containing Carbopol (C974P) and poloxamer (P407) showed complete release (100%) of curcumin after 8 h from the formulation, following time-dependent anomalous release behavior. Further, the cytotoxicity potential of the formulation was conducted on two carcinoma cells (FaDu and Cal27) and oral keratinocytes (FNB6), revealing an increase in the cytotoxic effects of Cal 27 and a decrease in cytotoxic effects in healthy cells [44].

Though solvent casting is a simple technique for producing buccal films, it is important to consider some of the hidden drawbacks:

- Solvent casting is a multistep process, which brings variation in the final product for each batch;
- Air entrapment is a crucial flaw in this process, which leads to dose variation;
- Application of organic solvent during the production process, because solvent removal from the buccal film and subsequent disposal is a tedious process.

Hot-melt extrusion could be an effective alternative for the solvent casting method, which provides a solvent-free, continuous manufacturing one-step process [62].

#### 3.2.3. Hot-Melt Extrusion

In the mid of 19th century, hot-melt extrusion (HME) was first introduced for the insulation of electric wires and became a widely used technique in the plastic industries. The major difference between the extruders used for the plastic industry and the pharmaceutical industry is the regulatory requirement of extruder contact parts. Because the contact parts are usually corrosive, reactive, or absorptive with the formulation. Thus, all contact surfaces are coated with stainless steel to provide non-corrosive, non-reactive features. HME is a suitable alternative for the solvent casting method. In the HME process, the raw materials (such as nutraceuticals/ bioactive compounds, mucoadhesive polymers, plasticizers) are transported through the rotating screws under elevated temperature through a die into a product of uniform shape. The hot-melt extrusion process is a four-step process [63]:

1. Feed the formulation ingredients and bioactive compounds to the extruder through a hopper;
2. Mixing, grinding, and kneading;
3. The molten ingredient conveyed to the through the rotating screw;
4. Extrusion through the die and mold the desired shape.

**Figure 5:**
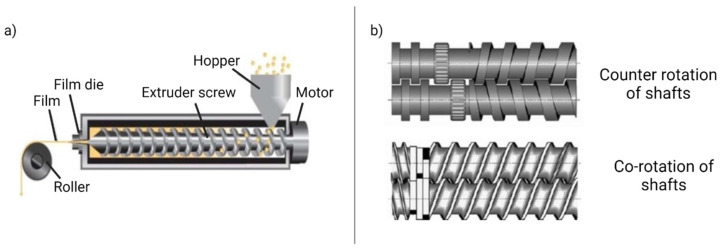
Hot-melt extrusion technique. (**a**) Production of buccal film; (**b**) counter-rotation and co-rotation of shafts (Reprinted with permission from Karki et al. 2016 [57]).

The extruder screw contains one or two rotating screws, which can provide co-rotating or counter-rotating options. Figure 5 depicted the hot-melt extrusion process equipped with a hopper, extruder screw, film die, and roller. HME process operates in the complete absence of solvents, and the therapeutic compounds with other ingredients are in the molten stage to obtain the homogeneous mixture. Thus, HME is not recommendable for heat-sensitive bioactive compounds.

### 3.3. Printing Technology

In the previous film production techniques (solvent casting and HME), therapeutic compounds, mucoadhesive polymers, and other excipients need to be mixed prior. Such premixing of ingredients with polymer matrix might produce solid amorphous dispersion. If the therapeutic compound is super-saturated in the polymer matrix, then there is a chance of phase separation during storage by crystallization. Further, crystallization could alter the dissolution of the therapeutic compound from the film and its mechanical properties [64]. The printing technique (also called inkjet printing) is a non-contact approach, where the ingredients are sprayed to create 2D and 3D structures. Broadly, inkjet technology can be classified either as continuous inkjet printing (CIJ) or drop-on-demand (DoD) printing. In CIJ, the liquid stream is passed through the orifice and piezoelectric transducer behind the nozzle “steer” the droplets and create a printed pattern. In DoD printing, the liquid ejects from the nozzle only when the drop is required [65].

Printing a mucoadhesive buccal film is an innovative platform to produce the formulation for the individual’s requirements and provide customized formulations and personalized medication at the point of care [66]. Ref. [67] reported the application of five different 3D printing techniques in the pharmaceutical sector: fused deposition model (FDM), binder jet printing, stereolithography, selective laser sintering, semi-solid extrusion. Two-dimensional printers are limited to X and Y direction printing; therefore, the carrier (sheet containing mucoadhesive polymers and other excipients) is pre-produced and holds/sorb the deposited ink in a predefined pattern, whereas 3D printers enable an additional Z direction by creating layer-by-layer structure and produce a 3D dosage form.

#### 3.3.1. Inkjet Printing (2D)

Inkjet printing (IJP) is a versatile, relatively inexpensive method of printing to produce dosage forms with remarkable accuracy and is suited for manufacturing low-dose medication and rapid production. Lord Rayleigh first described the mechanism by which the liquid stream is breaking up into droplets [68]. In the IJP technique, ink droplets (containing bioactive compound/nutraceuticals) jetted through a nozzle to create a pattern of dots on a given substrate (containing mucoadhesive polymers). This technique can be distinguished as continuous (dispense a continuous stream of droplets) and drop-on-demand mode (release droplets when required). Further, the IJP technique is equipped with single or multiple nozzles with a transducer, and the two main technologies employed for the IJP include: piezoelectric inkjet (PIJ) and thermal inkjet (TIJ) printing. For more detailed working principles on the piezoelectric and thermal inkjet printers, readers can refer the Edinger’s review manuscript [69].

#### 3.3.2. Flexographic Printer (2D)

**Figure 6:**
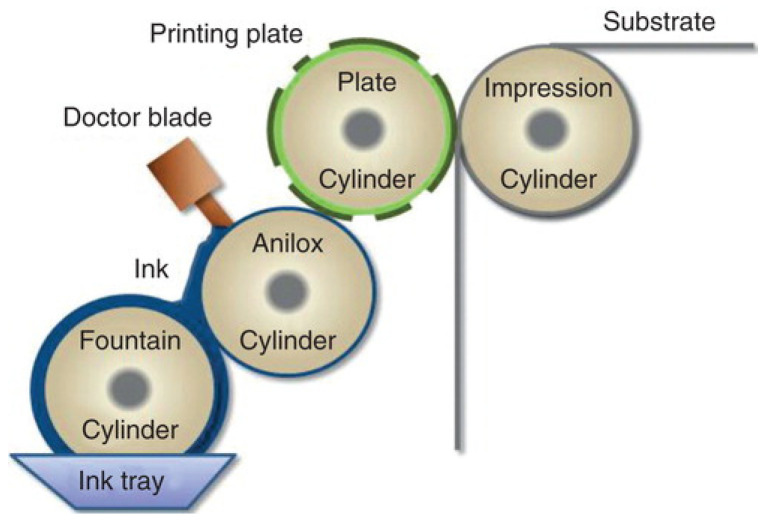
Flexographic printing process of the mucoadhesive film (Reprinted with permission from Kolakovic et al. 2013 [72]).

Flexographic printers (roll-to-roll printer) is most widely used for printing newspapers and magazines due to its flexible substrate options including paper, plastic, acetate film, and foil. The schematic representation of flexographic printing techniques is given in Figure 6. For film formulation, the pharmaceutical ink is loaded to anilox rollers to provide a measured amount of ink to the printing rollers. However, the use of flexographic printing is difficult in the pharmaceutical sector due to the requirement of organic solvent (in higher ratio) for drug solubilization and the inherent risk of precipitation and activity loss [70,71].

#### 3.3.3. Fused Deposition Modeling (3D)

3D printing is a digital process that involves the construction of a complex solid matrix by layer-by-layer structure and binds them as a single matrix either by phase transition or chemical reactions. Three-dimensional printing in food allows the user to design, tailor their nutritional need, and fabricate their food with customized shape, color, and flavor [73,74]. Several 3D printing technologies have been developed, including fused deposition modeling (FDM), laser sintering, laser-assisted bioprinting, and micro-extrusion technique. Fused deposition modeling is showing an extensive application in food industries, as this technique works similar to hot-melt extrusion with the aid to control /direct the position of the extrusion nozzle. In this technique, feedstock containing thermoplastic material and the therapeutic compound is melted and extruded through the nozzle at predetermined patterns to produce sequential layers of material [75].

**Figure 7:**
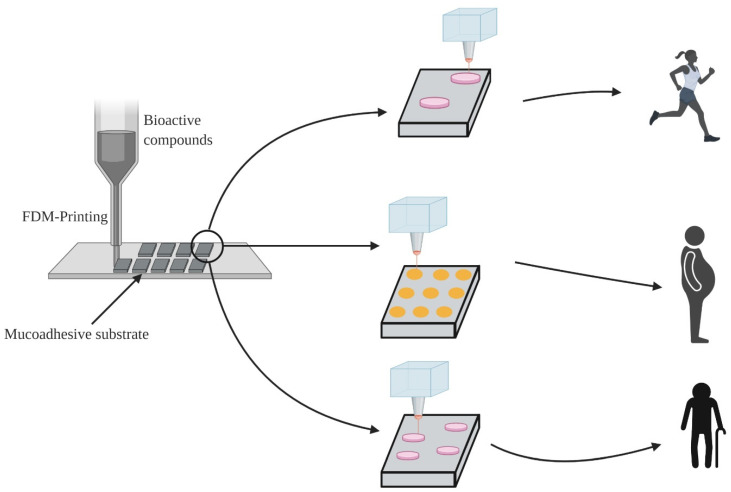
Personalized formulation of nutraceuticals for individuals using fused deposition models (created with Biorender.com (accessed on 11 June 2021)).

The process of 3D printing initiates from creating a digital template through 3D drawing tools such as Autocad Fusion 360 (Free for academic institutions), FreeCAD (OpenSource), and upload the model file in stl format to the commercial 3D printer. A computer-aided design (CAD) program moves the nozzle head within the x- and y-axes and the nozzle platform moves vertically (z-axis), creating 3D structures by fusing the layers. The critical parameters for 3D FDM include the speed of the nozzle, operating temperature, material density, and height of the layers [76]. Eleftheriadis et al. [77] proposed a new approach for buccal film formulations by combining IP and FDM technologies, whereas the dose accuracy and personalized dose were achieved by inkjet printing (IP), and mucoadhesive substrate of precise dimension was fabricated using FDM technique (see Figure 7). Such manufacturing facility will provide a key benefit for the in-situ manufacturing of mucoadhesive buccal films at the points of care. For instance, the nutraceutical requirement for athletics is different from an elderly person. The concept of personalized formulation helps to provide nutraceutical dose for individuals to meet their requirement.

### 3.4. Buccal Wafers

Buccal wafers are highly porous structured solid formulations produced by freeze-drying the polymer gels in dispersion form or solution. Due to their porous structure, wafers can easily disintegrate than conventional formulations. Many pharmaceutical and nutraceutical wafers available in the market are fast-disintegrating formulations, and the wafers for buccal administration are still in clinical trials, and there are no commercial products [78]. Boateng et al. [79] compared the release characteristics between freeze-dried wafers and solvent-cast film formulations. Scanning electron microscopy revealed the porous architecture with an interconnecting network for the wafers and a non-porous dense continuous sheet for the film. Interestingly, both wafer and film formulations followed sustained release behavior initiated by matrix swelling and drug diffusion through the swollen matrix. Later, the same group Boateng et al. [80] compared the release characteristics of wafer and films for the insoluble drug. Usually, formulation of low soluble drug exhibits erosion behavior due to the presence of a drug in the hydrated layer near the eroding front [81]. Surprisingly, the authors found that the sustained release characteristics were similar to the water-soluble drugs due to the involvement of factors other than solubility and mass transfer phenomenon. Thus, the release of the therapeutic compound from the buccal formulation depends on the physical properties (hydration, swelling, and erosion properties), amount and type of excipients, polymer grade, and its hydration characteristics. Later, Szabó et al. [82] developed vitamin B12 wafers and solvent-cast films by varying the proportion of mucoadhesive polymer Carbopol (CP). Release from wafers shows the partial diffusion from the swollen matrix and water-filled pores in the formulation. In addition, it is important to note that increasing mucoadhesive polymer (Carbopol), freeze-drying process, and storage decreased the rate of drug release from the mucoadhesive formulation. Because Carbopol is insoluble in the dissolution medium (phosphate buffer) and the authors revealed the swelling behavior of wafers is due to the COOH group hydrated by forming hydrogen bonds by absorbing water. Further, freeze-drying process results in porous network and increasing surface area of the polymeric system to swell when contact with the dissolution media and forming a rate controlling barrier for the drug release.

## 4. Mucoadhesive Polymers

Although diffusion of therapeutic compounds through the mucus is complex and controlled by many variables, including the size, charge, and wettability of the drug, it is required to analyze the most influencing parameter for the mucoadhesive property. Based on the mucoadhesion theories, readers can understand that the mucoadhesive property can be tailored by altering the interaction between polymer and mucosal surface. Mucoadhesive polymers are employed in pharmaceutical formulations since 1947 when Scrivener and Schantz [83], was trying to formulate a penicillin delivery system using gum tragacanth and dental adhesive powders. Later, the potential application of various polymers (e.g., sodium alginate, sodium carboxymethylcellulose, guar gum, hydroxyethylcellulose, methylcellulose, poly(ethylene glycol) (PEG), retene and tragacanth) found to exhibit mucoadhesive properties [84].

The main intention of mucoadhesive dosage formulation is to facilitate residence time of drug at the absorption site, provide sustained release characteristics and minimize the exposure of drug at various sites of the body [85]. Mucoadhesive polymers play a crucial role in extending the residence time of dosage form in the buccal cavity, which is achieved by the hydrophilic properties of the polymer. Both charged and non-ionic functional groups showed excellent mucoadhesive properties through their strong hydrogen bonding with the mucosal surface. Ideal polymer characteristics for mucoadhesive formulation should possess the following features [86,87]:

⮚ Strong hydrogen bonding groups, e.g., carboxyl, hydroxyl, amino- and sulfate groups;
⮚ Strong anionic or cationic functional groups;
⮚ Possess high molecular weight;
⮚ Surface tension to induce spreading into mucus layer;
⮚ High chain flexibility;
⮚ Capacity to load bioactive compounds;
⮚ Swell upon hydration;
⮚ Interact with mucus for adequate adhesion;
⮚ Provide controlled release of bioactives from the formulation;
⮚ Should be biologically degradable.

**Figure 8:**
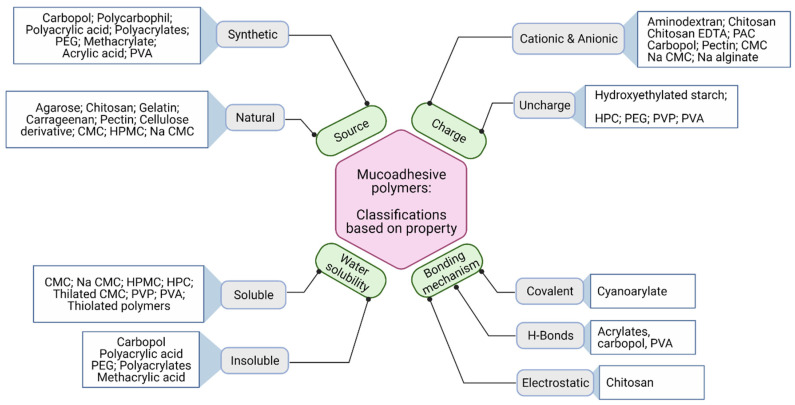
Classification of mucoadhesive polymers (Reprinted with permission from Mansuri et al. 2016 [92]).

Researchers classified the mucoadhesive polymers based on their origin (natural/synthetic), aqueous solubility, site of mucosa (buccal/ocular/nasal), or their chemical structure (cellulose/polyacrylates). Laffleur and Bernkop-Schnürch [88] classified them based on their binding mechanism with the mucosa: non-covalent binding polymers (mechanism of adhesion is due to the polymer’s surface charge) and covalent binding polymers (generate a covalent bond between mucus layer and polymer). In this review, the application of starch and chitosan polymers was described elaborately because of their frequent application in food science. Further, readers can refer following manuscripts [89,90,91] for detailed descriptions of the mucoadhesive polymers. Figure 8 summarizes the classification of mucoadhesive polymers based on their origin, charge characteristics, solubility, and bonding mechanisms.

### 4.1. Starch Buccal Films

Great attention to GRAS polymers made the researchers find an alternative for synthetic polymers because most synthetic polymers were synthesized from petroleum-based raw materials and showed a serious environmental problem. Biodegradable polymers from natural resources are of great interest because of their sustainability and related environmental benefits. Starch is considered a promising alternative due to its low cost, sustainability, and complete biodegradability [93]. The starch of different sources and different blends of copolymers were successfully employed for the mucoadhesive formulation. However, the native form of starch limits its application in the mucoadhesive systems due to its poor film-forming properties and semi-crystalline structure. Starch modification (performed by enzymatic, chemical, and physical treatment) is a suitable option for improving the mucoadhesive properties. Okonogi, Khongkhunthian, and Jaturasitha [93] revealed the amylose content in the rice starch has a significant effect on the mucoadhesive property of buccal films. Authors developed buccal films from chemically modified carboxymethyl rice starch (for two rice varieties) with an extreme difference in amylose content and different levels of crystallinity. Buccal films from low amylose content showed the halo patterns, which indicates the destruction of crystalline structure into amorphous form, whereas high amylose buccal films showed crystalline peak. In addition, low amylose films exhibited poor mucoadhesive strength (137.1 ± 5.1 kg m^−2^) than the high amylose film (191.5 ± 6.2 kg m^−2^). This increase in mucoadhesive strength for the high amylose buccal film is due to the formation of stiff network strands and entrapping more water in the network pores, which strengthens the bond between buccal film and mucosal surface. In a recent study, Miksusanti et al. [94] studied the mucoadhesive property of buccal films developed using chitosan and modified tapioca starch complex. Authors found that the developed buccal films can stick to the mucosal surface for 320 min owing to the hydrophilic group of tapioca starch and electrostatic interaction between chitosan (+ve charge) and mucin glycoprotein in the mucosa (−ve charge).

Few studies were investigated the physical modification of starch for improving its mucoadhesive properties. Recently, Soe et al. [95] applied different mechanical forces such as friction, collision, impingement, shear through ball milling to modify the starch structure (modified glutinous rice starch-MGRS). Authors developed buccal tablets using MGRS as mucoadhesive polymer and compared the mucoadhesive capabilities with commercial hydroxypropyl methylcellulose (HPMC) and sodium carboxymethylcellulose (NaCMC) tablets. HPMC and NaCMC are well-known mucoadhesive polymers following non-covalent binding mechanisms. Mucoadhesive strength was measured by calculating the force required to detach the tablets from porcine esophageal mucosa. Mucoadhesive strength of the buccal tablets was in the order of HPMC < MGRS < NaCMC. The native glutinous rice starch was not showing any mucoadhesive properties, and the modification improved the mucoadhesive properties with a detachment force of 0.2 N. The amorphous structure and improved hydration and swelling property of MGRS attributed to its mucoadhesive properties.

### 4.2. Chitosan

**Figure 9:**
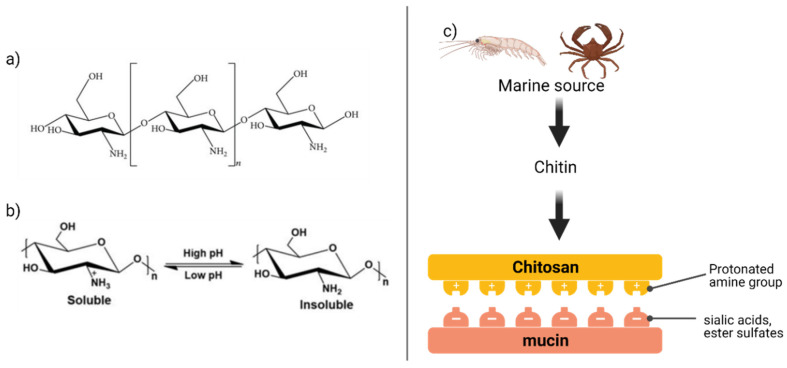
(**a**) chemical structure of chitosan molecule, (**b**) soluble characteristics, (**c**) production process of chitosan from marine source.

Chitosan (CS) is widely used in mucoadhesive dosage form, composed of *N*-acetyl-d-glucosamine and d-glucosamine, and its units are linked by 1-4-β-glycosidic bonds. CS is derived from chitin (abundant natural polysaccharide) by the deacetylation process. CS is a biocompatible and biodegradable polymer with interesting biological properties such as wound healing and antimicrobial properties. The presence of -OH and -NH_2_ groups facilitate the hydrogen and covalent bonding of chitosan (chemical structure is shown in Figure 9. At low pH (<6), these amino groups undergo protonation (addition of hydrogen proton) and make the chitosan as cationic (positively charged) macromolecules. On the other hand, mucins are negatively charged due to the presence of sialic acids and ester sulfates, which easily attract the CNs by strong electrostatic interactions [29]. Further, CN can transiently open the tight junctions and promote the paracellular transport of therapeutic compounds, but the signaling mechanism from chitosan to the tight junctions remains unclear. Hsu et al. [96] first proposed that chitosan activates the integrin receptors on cell membranes. Integrins are cell surface receptors comprising α/β heterodimeric complexes and interact with the signaling proteins. The direct interaction between the CN and integrin receptors led to the conformation change of integrin receptors, clustering along the cell border and initiating the cascade of tight junction openings. Authors confirmed the electrostatic interaction between chitosan and integrin through the molecular dynamic simulation studies and revealed -NH3+ groups of chitosan and the -COO- groups of integrin forms a chitosan-integrin complex.

The pKa value of the CS amino group is 6.5, which makes the CS chain protonated at acidic pH and deprotonated at neutral pH. However, the physiological pH of an intestinal segment is 6–7.4 [97], which reduces the ability of CS to open the tight junction of the intestinal segment and confined the absorption [98]. Extending the application of CS in the intestinal tract through chemical modification is a great opportunity to deliver the nutraceutical formulation. The target sites for CS are amine and hydroxyl groups. Such chemical modification increases the biocompatibility of CS and water-solubitliity at the physiological range (6.8–7.2) [99]. Further, chitosan possesses amenable amine (NH) and hydroxyl functional groups, which makes it possible to carry out chemical modification without disturbing the degree of polymerization. DP is defined as the number of repeating monomer units in the polymer, and it is calculated as the ratio of the molecular weight of the polymer to the molecular weight of the repeating monomer. Various chitosan derivatives, including carboxymethyl chitosan, trimethyl chitosan, glycol chitosan, and methylpyrrolidinone chitosan, were reported for improved retention on the mucosal surface [29]. Carboxymethyl chitosan is one of the most popular chitosan derivatives in the food and pharmaceutical industries. Carboxymethylation is targeted to occur at C-6 hydroxyl groups or the amine group [100]. Dekina et al. [101] retained the therapeutic activity of lysozyme in mucoadhesive films for three years using gelatin and carboxymethyl cellulose polymers. These polymers have the ability to form ionic or hydrogen bonds with bioactive compounds and provide non-covalent interaction with a lesser degree of affecting the enzyme structure. The prepared mucoadhesive films able to release 75% of the enzymatic activity after incubating for 30 min in Na-phosphate buffer solution, and the maximum enzymatic activity was maintained for 90–180 min.

**Figure 10:**
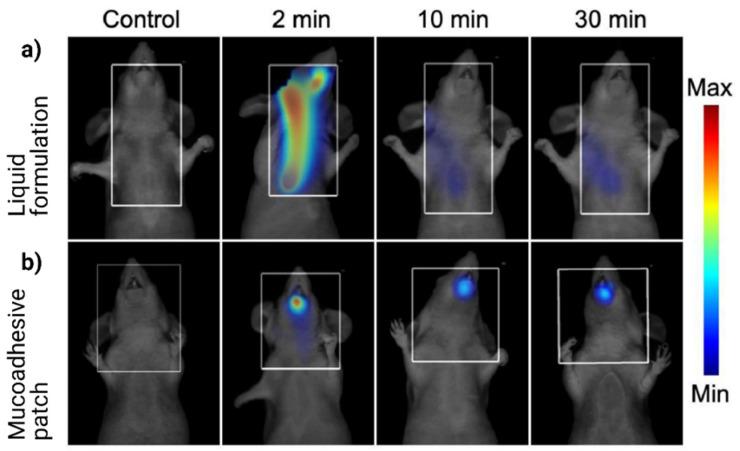
Fate of ovalbumin protein in conventional and mucoadhesive formulation: (**a**) rats administered with a liquid formulation of ovalbumin protein, (**b**) rats administered with ovalbumin mucoadhesive patch (Reprinted with permission from Paris et al. 2021 [102]).

In a recent study, Paris et al. [102] developed a mucoadhesive patch to deliver the protein using oppositely charged polyelectrolytes, chitosan, and hyaluronic acid. Authors visualized the distribution of ovalbumin from the conventional and mucoadhesive formulation using in vivo tomography analysis of protein residence time in the tongue. When the rats were administered with liquid ovalbumin formulation (conventional), the OVA was distributed along the digestive tract within 2 minutes after administration (see Figure 10), whereas mucoadhesive patches were showing a strong signal in the mouth for 30 min. Ovalbumin was penetrated rapidly into sublingual epithelium due to the chitosan polymer, which permeabilizes the tissue and facilitates the passive diffusion of proteins. Thus, 30-minute signals after administration with protein patch were decreasing its signal due to the protein clearance from the patch and uptake by antigen-presenting cells.

## 5. Conclusions

The mucoadhesive formulation for delivering bioactive compounds has a promising future in the food and nutraceutical industries. It is possible to use as the delivery system for poorly soluble bioactive compounds. Mucosal sites are easy to access, easy to remove, avoid enzymatic degradation, and provide rapid, controlled delivery of bioactive compounds for both local and systematic applications. Mucoadhesiveness depends on the structure, surface charge, hydration rate, molecular weight, surface tension, and concentration of polymers. Recently, thiolated polymers and natural polymers are widely investigated for their mucoadhesive properties. In addition to the conventional formulation technique, electrospinning and electrospraying techniques are receiving great attention to impart the nanofiber in mucoadhesive systems. The beauty of mucoadhesive formulation is the ability to provide a personalized formulation with a combo of nutraceuticals, and it should be explored further in commercial aspects. At the same time, it is important to note that mucoadhesive formulations are entirely new to consumers. It is necessary to educate them with a science-based approach. A thorough understanding of the oral mucosa, keratinized and nonkeratinized epithelium, mucoadhesive mechanisms, formulation techniques, and polymer interactions are necessary to formulate a novel mucoadhesive nutraceutical formulation.

## Figures and Tables

**Table 1:** Thickness and protein content of oral mucosa collected from the healthy volunteers (Reprinted with permission from Pramanik et al., 2010 [13]).

Saliva/Mucosal Surface	Mucosal Surface Condition	Thickness of Mucosal Fluid (μm)	Protein Concentration (mg mL^−1^)
Unstimulated whole-mouth saliva	n/a	n/a	3.07 ± 0.27
Anterior hard palate	Wet	9.6 ± 3.0	22.0 ± 5.5
Buccal mucosa	Wet	39.5 ± 7.4	7.1 ± 0.6
	Dry	17.1 ± 3.4	19.6 ± 7.4
Anterior tongue	Wet	54 ± 5.8	3.3 ± 0.7
	Dry	12.3 ± 2.2	12.5 ± 2.6
Lower labial mucosa	Wet	20.8 ± 2.5	22.2 ± 4.3
	Dry	6.0 ± 0.6	41.3 ± 13.5

**Table 2:** Challenges associated with the mucoadhesive dosage formulation.

Challenges	Description and Impact
Mucosal structure	It varies at different regions in the oral cavity, and the mucosal epithelial barrier act as a barrier
Saliva flow	The continuous secretion and flow of saliva may detach the formulation base
Variation	Mucosal surface vary person to person attributed to the tongue movements and variation in saliva secretion amount
Application region	Surface area available for the mucoadhesive formulation is very limited, which affects the loading capacity
Comfortability	The design of mucoadhesive formulation ensures easy installation/removal and accounts for the consumers’ comfortability

**Table 3:** Recent application of mucoadhesive dosage form for nutraceutical ingredients.

Sl. No	Bioactive Compound	Mucoadhesive Polymer	Mucoadhesive Formulation	Study Objective	Study Method	Research Findings	Reference
1	Zinc sulfate	Carbopol 940 + sodium alginate	Tablets	Zinc sulfate for the treatment of recurrent aphthous stomatitis (RAS)	Human clinical trial conducted with 46 participants having RAS	Conducted clinical trial with mouth ulcer patients, and authors found that the zinc tablets can reduce the pain, diameter of ulcer wounds and its inflammation, and accelerates the recovery time of ulcer.	[37]
2	Chitosan	Polyurethane + chitosan	Films	Chitosan mucoadhesive film for the treatment of RAS	Human clinical trial. 72 participants were recruited for the study and conducted data analysis with 66 subjects	Chitosan film promoted the healing of RAS, and the pain score was significantly reduced from day 4 to day 6. Chitosan films shielded the ulcer from external stimuli and thereby reduced the related pain from the ulcer region.	[38]
3	α-mangostin	Chitosan + alginate	Hydrogel films	α-mangostin hydrogel film for the treatment of RAS	In vitro release and mucoadhesive study in mouse mucosa	Chitosan +alginate +α-mangostin hydrogel films were adhesive toward the mouse mucosa for 46.7 min, and showing burst release characteristics, which is necessary for the treatment of RAS.	[39]
4.	Ginger extract	Tragacanth gum	Films	Ginger extract for the treatment of RAS	Clinical study in 15 patients	The mucoadhesive ginger formulation can relieve the pain of RAS patients; however, there is no statistical difference in the ulcer diameter, healing time with placebo.	[40]
5.	Curcumin	Hydroxypropyl methylcellulose + glycerin	Patches of 2 × 3 cm containing 2% curcumin	Curcumin for the treatment of oral submucous fibrosis (OMSF). OSMF is a chronic inflammatory, and immune-mediated disease occurs commonly by chewing arecanuts	Forty patients with OMSF in two groups. One group of patients was administered with curcumin gel and another group was administered with curcumin mucoadhesive patches	All patients were relieved from a burning sensation in the oral cavity, and the patients can open the mouth by 5.9 ± 2.00 mm. The curcumin patches were easy to apply and provided a non-invasive mode of treatment for OSMF.	[41]
6.	Curcumin	Zein + beta-cyclodextrin	Curcumin loaded nanoparticles	Develop Mucoadhesive zein NPs for curcumin buccal delivery	Ex vivo study. Mucoadhesive properties were conducted with buccal mucosa from freshly killed pigs	Curcumin permeation study revealed the highest curcumin permeation for zein cyclodextrin mucoadhesive formulation than the curcumin nanoparticles.	[42]
7	Curcumin	Gellan gum + Pectin	Films	The efficiency of gellan gum and pectin mucoadhesive formulation for delivering curcumin	In vitro release kinetics and disintegration	The mucoadhesive film was not disintegrated after 24 h exposed to simulated saliva. That means the film can remain in the target site for a prolonged time and release the therapeutic compounds. In vitro release study showed that there was an initial burst release till the first 10 min of exposure and then the release rate lowered up to 60 min of the test. Fast swelling of the film and rapid liquid uptake attributed to initial burst release behavior.	[43]
8	Curcumin	Poloxamer 407 + Carbopol 974P	Films	Develop nanostructured curcumin incorporated in films to target oral squamous cell carcinoma	In vitro release study, and ex vivo mucosal permeation in the porcine oral mucosa	A complete release of curcumin after 8 h exposure in the in vitro simulated condition, which makes it a suitable formulation for a buccal application.	[44]
9	Curcumin	Poly (L-lactic acid)	Patch	Develop mucoadhesive patch containing curcumin nanofibers using electrospinning techniques	In vitro release study and ex vivo adhesive properties in porcine buccal mucosa	Curcumin patch showed the least adhesion force of 0.14 ± 0.01 N, attributed to the non-mucoadhesive characteristics of polymer PLLA.	[45]
10	Resveratrol	Chitosan + Zein	Nanoparticles	Effectiveness of chitosan-coated zein nanoparticles for the oral delivery of resveratrol	In vitro mucoadhesion study in mucin solutions	Particle diameter was increasing with increasing mucin concentration as more mucin adsorbs on the nanoparticle surface. Thus, chitosan-coated nanoparticles were showing a higher increment in size than the uncoated particles.	[46]
11	Resveratrol	Hydroxypropyl cellulose + ethyl cellulose	Films	Optimize the polymer concentration on the mucoadhesive strength, swelling, and in vitro release	In vitro release study and ex vivo permeation study with goat buccal mucosa	Ex vivo permeation study revealed that there was a restriction in resveratrol permeation due to the low wetting and hydration of polymer matrix. In addition, the low aqueous solubility of resveratrol limited the release and penetration characteristics.	[47]
12	Peanut skin extract	Gelatin + hydroxypropyl methylcellulose	Films	Develop polyphenol enriched films using the casting technique	In vitro release technique	Mucoadhesive films followed the initial burst release of phenolic content about 1.2 mg gallic acid equivalent, attributed to the hydrophilic polymer, hydroxypropyl methylcellulose.	[48]
13	Pomegranate fruit extract	Carboxymethyl cellulose	Films	Produce multilayered oral films using printing techniques incorporating phenols	In vitro release technique	The release of polyphenols from the films showed a Fickian diffusion pattern, and the phenolic compounds were stable for 196 days at room temperature.	[49]
14	Vitamin B-12	Chitosan + Polyvinyl alcohol	Films	Develop vitamin B-12 mucoadhesive hydrogel films	In vivo pharmacokinetic study with rabbits	Compared the release of vitamin B12 from the buccal films and commercial Neuroton I.M. injection. The area under the curve (AUC_0–8h_) showed a 1.5-fold increase in bioavailability from the buccal film compared with the I.M. injection.	[50]
15	Vitamin B12	Hydroxypropyl methyl cellulose + Carbopol + chitosan	Tablets	Develop buccal tablets of vitamin B12 and improve the oral bioavailability	In vivo pharmacokinetic study with rabbits	Rabbits injected with vitamin B12 (intramuscular) showed rapid release till 15 min at a maximum concentration of 109.29 ± 9.39 pg/mL and gradually decreased to 43.23 ± 2.034 pg/mL at 30 min. Rabbits administered with buccal tablets were released vitamin B12 in a sustained release manner and showed a 2.7-fold increase in bioavailability compared to the I.M. injection.	[35]
16	Vitamin K	Labrasol + Transcutol	Self-nano emulsifying lyophilized tablets	Improve the oral bioavailability of vitamin K	Human clinical trial. A group of volunteers administered buccal tablets and another group with intramuscular injection	Pharmacokinetic study in human volunteers revealed the buccal tablets enhanced vitamin K absorption and relative bioavailability. Interestingly, there was no significant difference in the vitamin K in systemic circulation for the two groups of volunteers.	[51]
17	B-complex vitamins: thiamine hydrochloride (THCl) and nicotinic acid (NA)	Propylene glycol	Films	Develop vitamin B-complex buccal films by inkjet printing technique	In vitro technique	Both vitamins are released within 10 minutes from the buccal film. For increasing vitamin content, there was an increase in permeation across the cellulosic membrane.	[52]
